# Microbial profile and antimicrobial resistance patterns of ocular infection pre/during and post the COVID-19 pandemic

**DOI:** 10.3389/fmed.2025.1594150

**Published:** 2025-09-04

**Authors:** Min Du, Ying Zhang, Tian Xin, Yan Liu, Shumei Zhang

**Affiliations:** ^1^Department of Hospital Infection Management, Jinan Second People's Hospital, Jinan, China; ^2^Department of Clinical Laboratory, Shandong Second Provincial General Hospital, Jinan, China; ^3^Department of Operating Room, Jinan Second People's Hospital, Jinan, China; ^4^Department of Ophthalmology, Jinan Second People's Hospital, Jinan, China

**Keywords:** COVID-19, ocular infection, antimicrobial resistance, type I incision, infection incidence

## Abstract

**Background:**

This research investigated the distribution characteristics and antimicrobial resistance patterns of pathogenic microorganisms in patients with ocular infection before/during and post the coronavirus disease 2019 (COVID-19) pandemic.

**Methods:**

This retrospective study, conducted at the Second People’s Hospital of Jinan, Shandong, China, analyzed the microorganism cultures of specimens (eye secretions, anterior chamber fluid, and vitreous body) obtained from patients with ocular infection (including ocular trauma, endophthalmitis, keratitis, conjunctivitis, dacryocystitis, and blepharitis) pre/during (from May 2019 to January 2023, A group) and after (from February 2023 to November 2024, B group) the COVID-19 pandemic. The microorganism species was analyzed using a microbial identification instrument, and antimicrobial susceptibility testing was carried out using Kirby-Bauer (K-B) disk diffusion method.

**Results:**

465 and 319 strains of pathogenic microorganisms were obtained from specimens in A and B groups, respectively. The isolates of *Fusarium*, *Aspergillus* and other Filamentous fungi were significantly increased in B group, while no substantial difference was discovered in the isolates of gram-positive or gram-negative bacteria between these two groups. Increased resistances of *Staphylococcus epidermidis* to ampicillin (100%) and Penicillin G (100%), *Staphylococcus aureus* to ampicillin (100%) and penicillin G (100%), and *corynebacterium* to cotrimoxazole (90.0%) were observed in B group, in comparison with those of A group (65.5, 64.7, 26.9, 25.6 and 66.7%, respectively). The infection rate of type I incision of ocular surgery during the pandemic (0.018%) was substantially lower than that pre (0.07%) or post (0.06%) the prevalence.

**Conclusion:**

Changes in resistance patterns were observed after the COVID-19 pandemic, which might be influenced by relaxation of infection prevention and control (IPC) measures. These alterations might be also attributed to other factors, such us changes over time or the use of antibiotics. And further investigation was required to establish causality.

## Introduction

Ocular infections refer to common ophthalmic diseases induced by numerous microbial infections (1), which continue to be significant public health concerns worldwide (2). It has been certified that eye infections have a close bearing on many risk factors, including surgery, trauma, chronic nasolacrimal duct obstruction, dry eye, and contact lens wearing (3). If not properly treated, these infections may alter the normal structure of the eye, which results in visual impairment and even blindness (4, 5).

The dominant pathogens in ocular infections are gram-positive bacteria, followed by gram-negative bacteria and fungi (6). Among gram-positive bacteria, coagulase-negative staphylococci (*Staphylococcus epidermidis*, *Staphylococcus haemolyticus* and *Staphylococcus hominis*) are the most frequently separated species, followed by *Staphylococcus aureus* (7). Besides, increasing detections in *corynebacterium* and fungi have been reported in recent years (8, 9). Local use of antibiotics in ophthalmology is relatively limited, while aminoglycosides, penicillins, tetracyclines, fluoroquinolones, macrolides, phenicols and sulfonamides are the most utilized antibiotic classes for treating ophthalmic infections (10). Nonetheless, due to the increase of bacterial strains resistant to different antimicrobial agents, the treatment of ocular infections have become complicated (1). Hence, the altered antimicrobial resistance of microorganisms implicated in eye infections requires surveillance to guide the empirical therapy.

The emergence of severe acute respiratory syndrome coronavirus 2 (SARS-CoV-2) has triggered a pandemic called coronavirus disease 2019 (COVID-19), which has become the greatest worldwide public health threat of this century (11). A 5-year comparative study has reported the alterations of microbiological profiles in microbial keratitis before, during, and post COVID-19 (12). On the other hand, ophthalmic inpatients are always relatively simple, most of whom undergo selective surgery and receive systemic physical assessment with good health condition. Elderly individuals with acute severe COVID-19 infection are less in the department of ophthalmology. The surveillance of antimicrobial resistance in ocular pathogens may help to predict the shifting trends of drug resistance in ophthalmic patients, and provide a theoretical basis for antimicrobial selection in clinic.

In this study, the differences of pathogen distribution and antimicrobial resistance in specimens from patients with eye infections pre/during and post the COVID-19 epidemic were investigated retrospectively. Type I incision refers to a surgical incision that is sterile. Surgical incision infections include superficial incisional and deep incisional infections (13). Grade C healing in Type I incision indicates the situation that abscess occurs in sterile surgical incision (14). Here, we also explored the infection incidence in type I incision of ocular surgery during these periods. This study suggests that relax implementation of infection prevention and control post the COVID-19 epidemic may contribute to increased isolates of fungi, increased antimicrobial resistance, as well as the infection incidence in type I incisions.

## Methods

### Subjects

The clinical data of patients with ocular infection (including ocular trauma, endophthalmitis, keratitis, conjunctivitis, dacryocystitis, blepharitis) admitted to the Second People’s Hospital of Jinan in Shandong province from May 2019 to November 2024 were retrospectively collected. Specimens (eye secretions, anterior chamber fluid, and vitreous body) were obtained strictly according to the aseptic operating procedure. Then they were isolated and identified in the microbiology laboratory of the Department of Laboratory Medicine. This study was approved by the ethics committee of the Second People’s Hospital of Jinan in line with the Declaration of Helsinki (JNEYE20240654). The need for patient consent was waived due to the retrospective nature of the study. Patients were divided into two groups: A group (patients hospitalized pre/during the COVID-19 pandemic from May 2019 to January 2023), and B group (patients hospitalized after the pandemic from February 2023 to November 2024). Inclusion criteria: (1) clinically diagnosed diseases including ocular trauma, endophthalmitis, keratitis, conjunctivitis, dacryocystitis, and blepharitis; (2) clinical microbial culture specimens of eye secretions, anterior chamber fluid, and vitreous body. Exclusion criteria: (1) non-ocular biological specimens; (2) suspected contaminated strains; (3) non-ocular infectious diseases.

### Specimen collection, strain identification and antimicrobial sensitivity test

Eye secretions were gently wiped using a saline-soaked cotton swab. Besides, specimens of anterior chamber fluid and the vitreous body were taken intraoperatively. Subsequently, bacterial culture medium, fungal culture medium and enrichment culture medium were utilized to inoculate the specimens, followed by culture and separation. Afterwards, a microbial identification instrument (MicroScan autoSCAN-4, Dade Behring, Inc.) was used to identify the selected single colonies, followed by *in vitro* antimicrobial susceptibility test. Bacterial growth was detected on blood agar plates and chocolate agar plates at 37 °C for 24 h. For the detection of fungi, samples were cultured in Sabouraud medium at 30 °C for 7 d. Fungal species were identified based on morphological characters. Dominant strains were isolated and purified based on the morphological and staining changes of bacteria, obtaining pure culture of single strain for subsequent identification. The growth of miscellaneous bacteria was preliminarily identified by staining. The strains that were suspected to be contaminated were identified by assessing whether the initial growth point was on the inoculation line, and whether the strain was a common contaminated bacterium.

Antimicrobial susceptibility test was performed in all gram-positive and gram-negative bacteria cultivated in the specimens. Meanwhile, antimicrobial susceptibility test was carried out with Kirby-Bauer (K-B) disk diffusion method in line with Clinical and Laboratory Standards Institute (CLSI) guideline (CLSIM100) of the corresponding year. CLSI guideline is updated annually, and the criterion in our laboratory has been updated in time according to the guideline, to ensure accurate results. All experiments were performed strictly according to the National Guide to Clinical Laboratory Procedures. Calculation of infection incidence in type I incisionsThe surgical site infection incidence in type I incisions was calculated as follows: the number of people with surgical site infection in type I incisions/the number of type I incision operations during the same period ×100%.

### Statistical analysis

All data in this research were statistically analyzed with SPSS 22.0 software. Categorical variables were presented as frequencies and percentages, and were compared using Chi-square test. A *p*-value of < 0.05 was regarded as statistical significance.

## Results

### Distribution of pathogens

First, specimens including eye secretions, anterior chamber fluid and vitreous body were collected from patients with ocular infection for microorganism analysis. In this study, 465 and 319 strains of pathogenic microorganisms were obtained from specimens pre/during (A group) and after (B group) the COVID-19 pandemic, respectively. Among that, *Staphylococcus epidermidis*, *Staphylococcus aureus* and *Corynebacterium* were the top three gram-positive bacterial infections which accounted for 47.5, 16.8 and 11.6% in A group, and 44.2, 13.5 and 9.4% in B group, respectively. There was no obvious difference in isolates of gram-positive bacteria between these two groups. The top three pathogens among gram-negative bacteria infections were *Escherichia coli*, *Enterobacter cloacae* and *Aeromonas hydrophila* accounting for 1.1, 1.1% and 0. 9% in A group, and *Escherichia coli, Enterobacter cloacae* and *Pseudomonas aeruginosa* accounting for 0.6, 0.9 and 0.6% in B group, respectively. The difference in gram-negative bacteria isolates was not obvious between A and B groups. Additionally, *Fusarium*, *Aspergillus* and other Filamentous fungi were the top three pathogens in fungi, with 3.4, 2.2 and 2.6% in A group, and 7.2, 6.0 and 6.0% in B group, respectively. Notably, the isolates of *Fusarium*, *Aspergillus* and other Filamentous fungi were significantly increased in B group, compared with those of A group (Table 1).

**Table 1:** The isolates of *Fusarium*, *Aspergillus* and other filamentous fungi were significantly increased in patients with ocular infections after the prevalence of COVID-19.

Pathogen	A group (*n* = 465)	B group (*n* = 319)	*χ* ^2^	*p*
Gram-positive bacteria				
*Staphylococcus epidermidis*	221 (47.5)	141 (44.2)	0.842	0.359
*Staphylococcus aureus*	78 (16.8)	43 (13.5)	1.573	0.210
*Corynebacterium*	54 (11.6)	30 (9.4)	0.965	0.326
*Viridans streptococcus*	11 (2.4)	5 (1.6)	0.603	0.437
*Enterococcus faecalis*	9 (1.9)	2 (0.6)	1.491	0.222
*Enterococcus durans*	0 (0.0)	3 (0.9)	2.269	0.132
*Staphylococcus haemolyticus*	5 (1.1)	2 (0.6)	0.072	0.788
*Viridans streptococcus*	4 (0.9)	2 (0.6)	0.000	1.000
*Viridans streptococcus*	3 (0.6)	1 (0.3)	0.017	0.896
*Brevibacillus brevis*	2 (0.4)	1 (0.3)	0.000	1.000
Gram-negative bacteria				
*Escherichia coli*	5 (1.1)	2 (0.6)	0.072	0.788
*Acinetobacter lwoffii*	2 (0.4)	1 (0.3)	0.000	1.000
*Aeromonas hydrophila*	4 (0.9)	1 (0.3)	0.238	0.625
*Pseudomonas aeruginosa*	3 (0.6)	2 (0.6)	0.000	1.000
*Citrobacter koseri*	3 (0.6)	1 (0.3)	0.017	0.896
*Enterobacter cloacae*	5 (1.1)	3 (0.9)	0.000	1.000
Fungi				
*Fusarium*	16 (3.4)	23 (7.2)	5.686	0.017*
*Aspergillus*	10 (2.2)	19 (6.0)	7.692	0.006**
Other Filamentous fungi	12 (2.6)	19 (6.0)	5.676	0.017*
others	18 (3.9)	18 (5.6)	1.356	0.244

*p < 0.05, **p < 0.01.

### Antimicrobial resistance of pathogenic strains

Antimicrobial sensitivity testing displayed that the resistances of *Staphylococcus epidermidis* to ampicillin and Penicillin G were significantly raised post the COVID-19 prevalence (both 100%), in comparison to that before/during the pandemic (65.5 and 64.7% respectively). However, no statistical significance was observed in *Staphylococcus epidermidis* resistance to other antibiotics between the two groups, such as Ceftriaxone, Erythromycin, and Gentamicin (Table 2). Besides, *Staphylococcus aureus* isolates were resistant to ampicillin (100%) and penicillin G (100%) in B group, which were remarkably higher than those of A group (26.9 and 25.6% respectively). No obvious difference in the resistance of *Staphylococcus aureus* to other antibiotics was found between these two groups, such as Clindamycin, Ceftriaxone and Erythromycin (Table 3). Among *corynebacterium* isolates, higher resistance to cotrimoxazole (90.0%) was observed in the B group, compared with that of A group (66.7%). The difference of *corynebacterium* resistance to other antibiotics was not significant between A and B groups (Table 4).

**Table 2:** The resistances of *Staphylococcus epidermidis* to ampicillin and penicillin G were significantly raised post the COVID-19 prevalence.

Antibiotics	A group (*n* = 221)	B group (*n* = 141)	*χ* ^2^	*p*
Amoxicillin/Clavulanic acid	141 (63.8)	91 (64.5)	0.020	0.886
Ampicillin	145 (65.6)	141 (100.0)	61.374	0.000***
Clindamycin	97 (43.9)	77 (54.6)	3.962	0.047
Ceftriaxone	141 (63.8)	91 (64.5)	0.020	0.886
Erythromycin	169 (76.5)	112 (79.4)	0.435	0.510
Gentamicin	104 (47.1)	54 (38.3)	2.686	0.101
Levofloxacin	132 (59.7)	89 (63.1)	0.417	0.519
Moxifloxacin	9 (4.1)	12 (8.5)	3.103	0.078
Furantoin	1 (0.5)	0 (0.0)		1.000
Oxacillin	141 (63.8)	91 (64.5)	0.020	0.886
Penicillin G	143 (64.7)	141 (100.0)	63.432	0.000***
Rifampicin	6 (2.7)	2 (1.7)	0.204	0.652
Ampicillin/Sulbactam	147 (66.5)	92 (65.2)	0.062	0.804
Cotrimoxazole	125 (56.6)	70 (49.6)	1.657	0.198
Tetracycline	55 (24.9)	34 (24.0)	0.028	0.868

^***^p < 0.001.

**Table 3:** *Staphylococcus aureus* isolates were more resistant to ampicillin and penicillin G after the COVID-19 prevalence.

Antibiotics	A group (*n* = 78)	B group (*n* = 43)	*χ* ^2^	*p*
Amoxicillin/Clavulanic acid	19 (24.4)	16 (37.2)	2.226	0.136
Ampicillin	21 (26.9)	43 (100.0)	59.409	0.000***
Clindamycin	60 (76.9)	32 (74.4)	0.095	0.757
Ceftriaxone	19 (24.4)	16 (37.2)	2.226	0.136
Erythromycin	65 (83.3)	37 (86.0)	0.154	0.695
Gentamicin	22 (28.2)	13 (30.2)	0.055	0.814
Levofloxacin	23 (29.5)	12 (27.9)	0.034	0.854
Moxifloxacin	4 (5.1)	1 (2.3)	0.070	0.792
Furantoin	0 (0.0)	0 (0.0)	–	1.000
Oxacillin	22 (28.2)	14 (32.6)	0.251	0.616
Penicillin G	20 (25.6)	43 (100.0)	61.411	0.000***
Rifampicin	0 (0.0)	0 (0.0)	–	–
Ampicillin/Sulbactam	20 (25.6)	17 (39.5)	2.521	0.112
Cotrimoxazole	5 (6.4)	3 (7.0)	0.000	1.000
Tetracycline	14 (17.9)	3 (7.0)	2.763	0.096

^***^p < 0.001.

**Table 4:** Among *corynebacterium* isolates, higher resistance to cotrimoxazole was observed post the COVID-19 prevalence.

Antibiotics	A group (*n* = 54)	B group (*n* = 30)	*χ* ^2^	*p*
Clindamycin	50 (92.6)	24 (80.0)	1.839	0.175
Ceftriaxone	4 (7.4)	1 (3.3)	0.076	0.783
Erythromycin	47 (87.0)	27 (90.0)	0.003	0.960
Gentamicin	16 (29.6)	5 (16.7)	1.728	0.189
Penicillin G	7 (13.0)	4 (13.3)	0.000	1.000
Cotrimoxazole	36 (66.7)	27 (90.0)	5.600	0.018*
Tetracycline	15 (27.8)	3 (10.0)	3.620	0.057
Vancomycin	0 (0.0)	2 (6.7)	–	0.125

^*^p < 0.05.

### Infection incidence of type I incision in ocular surgery

We investigated the influence of COVID-19 pandemic on the infection incidence of type I incision in ocular operation. As shown in Table 5, the infection rate of type I incision during the COVID-19 prevalence (0.02%) was substantially lower than that pre (0.07%) or post (0.06%) the pandemic.

**Table 5:** Infection rate of type I incision was increased post the COVID-19 pandemic.

Group	Infection (*n*)	Type I incision (*n*)	Infection rate (%)	*χ* ^2^	*p*
A group	7	9,737	0.07	7.951	0.019*
B group	6	32,516	0.02^a^		
C group	9	15,206	0.06^bc^		

A, B, and C group: before, during, and post the pandemic. *p < 0.05. a: *p* = 0.021 compared to A group; b: *p* = 0.699 compared to A group; c: *p* = 0.039 compared to B group.

## Discussion

Evidence has noticeably demonstrated that during COVID-19 pandemic, effective implementation of infection prevention and control (IPC) interventions contributes to a significant decrease in health care-associated infection (15). In this study, we found significantly increased isolates of *Fusarium*, *Aspergillus* and other Filamentous fungi post the COVID-19 pandemic. Additionally, the resistance of several bacteria to antibiotics was increased after the pandemic, as well as the infection incidence of type I incision of ocular surgery. The present study proposes that relaxed IPC may have affected health care-related infection and antimicrobial resistance.

Antibiotics have good preventive and therapeutic impacts on bacterial disease and are extensively applied in the field of human medicine (including surgery, chemotherapy and infectious disease treatment) (16). On the other hand, rapid increase of antimicrobial resistance triggered by overuse of antibiotics has become one of the most challenging healthcare problems worldwide (17). With the help of antimicrobial stewardship program, China has made remarkable strides in controlling drug resistance over the past decades (18). Notably, bacterial coinfection and secondary infection are essential complications of COVID-19, which lead to antibiotic overuse during the COVID-19 pandemic (19, 20). The usage of antibiotics may lead to the variations in ocular surface microbiota which is composed of several microorganisms ranging from bacteria to fungus (21). An unbalanced microbiota can result in pathogenic microbial overgrowth (22). There is a retrospective case-note review of corneal scrape samples from microbial keratitis patients from January 2018 to December 2023 (12), which shows that gram-positive bacteria are the dominant pathogens in all periods, while there is no significant difference in their distribution and the most frequently identified organism is *Staphylococcus epidermidis*; Fungal infections are considerably increased in the post-COVID period, while *Fusarium* sp. is the most common fungus and its incidence is significantly increased; The most common gram-negative bacterium is *Pseudomonas aeruginosa*, and the incidence of *Pseudomonas aeruginosa* is substantially reduced in the post-COVID period. In our study, there was no substantial difference in the isolates of gram-negative or gram-positive bacteria. Interestingly, we found that the isolates of fungi including *fusarium*, *aspergillus* and other filamentous fungi were increased in specimens from patients with ocular infections after the COVID-19 prevalence. Potential affecting factors may be the following: strict health care policy and long lasting of management and control, long lasting of antibiotic use for hospitalized patients, and the usage of high-grade antibiotics for preventing severe pneumonia. Besides, the alterations of fungal pathogens may also be influenced by potential confounding factors (e.g., changes in antifungal prescribing practices, environmental factors).

The discoveries of a previous research have highlighted remarkable shifts in antimicrobial resistance patterns of uropathogenic bacteria during the COVID-19 pandemic, which present a general trend of decreased resistance of *Klebsiella pneumoniae* and *Escherichia coli* strains to several antibiotics during COVID-19; besides, reduced resistances of *Pseudomonas aeruginosa* strains to many antibiotics has been observed during COVID-19 (23). A multicenter retrospective study has evaluated the influence of the COVID-19 pandemic in the microbial profile and antimicrobial resistance patterns of bacteria isolated from cerebrospinal fluid specimens of patients with bacterial meningitis, which indicates a declining trend in resistance rates for coagulase-negative *Staphylococcus* and *Acinetobacter baumannii* to certain antibiotics after the pandemic; Conversely, the resistance to imipenem in *Acinetobacter baumannii* is increased (24). Here, post the prevalence of COVID-19, we identified increased *Staphylococcus epidermidis* resistance to ampicillin and Penicillin G, and *Staphylococcus aureus* resistances to ampicillin and penicillin G in specimens. The findings suggested possible exposure history to ampicillin and Penicillin G post the prevalence, resulting in selective proliferation of resistant strains and decrease of drug sensitivity. The resistance of cotrimoxazole, which is composed of sulfamethoxazole and trimethoprim, can be attributable to mutations in the genes encoding dihydropteroate synthase and dihydrofolate reductase (25). Here, the present study also identified elevated *corynebacterium* resistance to cotrimoxazole in specimens post the prevalence. Our discoveries indicated potential differences in the percentage of strains carrying resistant gene, or in the usage frequency and treatment course of cotrimoxazole, which contributed to alteration of cotrimoxazole sensitivity.

It has been reported that an information-assisted transparent supervision and multidisciplinary team model improves infection control and antimicrobial utilization in ophthalmology, and the incidences of surgical site infection for type I incision surgery (0.10%) and nosocomial infection (0.09%) both reduce to 0.00% (26). In the present study, we found that the infection incidence of type I incision in ocular surgery was decreased during the prevalence of COVID-19 compared to that pre or post the pandemic. Possible influencing factors may be the following. Firstly, during the pandemic, vigorous control measures have been implemented in China to limit the spread of COVID-19 (27). Most of the surgical areas in China were vacant during the outbreak, and elective surgeries were suspended within the specified period (28), which resulted in the growing backlog of untreated surgical diseases. Consequently, post the prevalence of COVID-19, numerous patients were hospitalized after the relaxation of prevention and control measures. Secondly, both adhering to hand hygiene guidelines and practicing hand hygiene are expected to reduce the risk of pathogen transmission between healthcare settings and patients (29). However, standard preventions such as hand hygiene and disinfections of the air and object surface could not be strictly implemented by medical personnels, due to their busy work. Finally, just after the COVID-19 pandemic, numerous individuals were infected with COVID-19 in the community. Some patients were in the acute phase of COVID-19 with unrelieved respiratory symptoms like cough, while some at the early stage were not fully recovered and had low immunity. This might be an important factor affecting postoperative incision infection. Further study is still needed to establish causality for interpreting higher infection incidence post COVID-19 prevalence. Our findings are of great significance for surgical patients and provide a basis for management of medical system.

In conclusion, in this study, we reported increased isolates of *Fusarium*, *Aspergillus* and other Filamentous fungi post the COVID-19 prevalence, in comparison with those pre/during the pandemic. Post the epidemic, elevated *Staphylococcus epidermidis* resistance to ampicillin and Penicillin G, *Staphylococcus aureus* resistance to ampicillin and penicillin G, and *corynebacterium* resistance to cotrimoxazole were found. Our study also uncovered an increase in infection incidence of type I incision of ocular surgery post the prevalence. This study suggests that IPC relaxation after the prevalence may influence drug resistance and infection of related pathogens. Other factors, such us changes over time or the use of antibiotics, might also be the reasons for these alterations. However, more investigation was needed to establish causality. On the other hand, there are limitations in the generalizability of the findings due to the study’s single-center design. Currently, direct evidences regarding the attribution of post-pandemic changes to surgical backlogs, relaxed hygiene, and COVID-19-related immunosuppression are still lacking. Besides, this study acknowledged potential confounding factors, such as changes in healthcare-seeking behavior, variations in antibiotic prescribing practices, or differences in patient demographics during the pandemic.

## Data Availability

The original contributions presented in the study are included in the article/supplementary material, further inquiries can be directed to the corresponding authors.
